# Impact of Lifestyle Modifications Along With Pharmacological Treatment of Heart Failure: A Narrative Review

**DOI:** 10.7759/cureus.81570

**Published:** 2025-04-01

**Authors:** Elizabeth Caroline Palaparthi, Priyanka K, Arockiamary Ignasimuthu, Gaoudam N, Nagasaikaran Sade, Naveen Bade, Bharath Kumar Jakka, Khyathi Krishna Gogineni, Anjaneyulu Dunde, Tambi Medabala, Panneerselvam Periasamy

**Affiliations:** 1 Department of Internal Medicine, Shasta Regional Medical Center - Prime Healthcare, Redding, USA; 2 Department of Obstetrics and Gynecology, Vinayaka Mission's Kirupananda Variyar Medical College and Hospitals, Vinayaka Mission's Research Foundation (Deemed to be University), Salem, IND; 3 Department of Medical Surgical Nursing, Faculty of Health Sciences, Villa College, Malé, MDV; 4 Department of Medical Surgical Nursing, Vinayaka Mission's College of Nursing, Vinayaka Mission's Research Foundation (Deemed to be University), Karaikal, IND; 5 Department of Internal Medicine, Apollo Institute of Medical Sciences and Research, Hyderabad, IND; 6 Department of Nephrology, Northeast Alabama Regional Medical Center, Anniston, USA; 7 Department of Internal Medicine, Baptist Medical Center South, Montgomery, USA; 8 Department of Stroke Medicine, Stepping Hill Hospital, Stockport, GBR; 9 Department of Physiology, Netaji Subhas National Institute of Sports, Sports Authority of India, Patiala, IND; 10 Department of Physiology, Government Erode Medical College and Hospital, Erode, IND

**Keywords:** heart failure, heart failure management, integrative approach, lifestyle modifications, pharmacological treatment

## Abstract

Heart failure (HF) remains a leading cause of morbidity and mortality worldwide. While pharmacological therapy is foundational, lifestyle modifications are increasingly recognised for their complementary role.

This narrative review explores the synergistic effects of lifestyle interventions, combined with pharmacological treatment, in HF management.

A literature search (2000-2025) was conducted using PubMed, Scopus, Cochrane, and Google Scholar, to identify studies on integrative approaches to HF care.

Lifestyle changes, such as dietary modification, exercise, weight management, smoking and alcohol cessation, and psychosocial support, enhance the efficacy of standard therapies, improve quality of life, and reduce hospitalisations. Integration with medications, like renin-angiotensin-aldosterone system (RAAS) inhibitors, beta-blockers, and sodium-glucose cotransporter-2 (SGLT2) inhibitors, shows improved neurohormonal balance, reduced inflammation, better endothelial function, and delayed cardiac remodelling. However, socioeconomic and cultural barriers challenge real-world implementation.

Combining lifestyle interventions with pharmacotherapy provides a holistic, patient-centred strategy for HF management. Future efforts should focus on personalised care, multidisciplinary teams, and policy support, to improve adherence and outcomes.

## Introduction and background

Brief overview of heart failure (HF) and its global burden

HF is a chronic and progressive cardiovascular condition characterised by the heart's inability to pump sufficient blood to meet the body's metabolic demands [1]. It is associated with significant morbidity and mortality. Recent epidemiological data indicate an increasing prevalence of HF due to ageing populations, rising rates of hypertension, diabetes, and obesity, and improved survival rates following myocardial infarction [2]. Approximately 64 million individuals are affected worldwide, contributing to soaring healthcare costs and a diminished quality of life [3].

Importance of an integrative approach combining pharmacological treatment with lifestyle modifications

Historically, pharmacological interventions have constituted the cornerstone of HF management [4]. Social determinants of health, including socioeconomic status (SES), access to healthcare, education, housing, food security, and environmental exposures, profoundly influence HF outcomes. Patients from underserved backgrounds often face structural disadvantages, such as inadequate insurance coverage, lower health literacy, unemployment, and mental health disorders, all of which exacerbate non-adherence, symptom progression, and hospital readmissions [5]. Further, lifestyle modifications, including dietary changes, structured physical activity, weight management, smoking cessation, and psychosocial interventions, are increasingly recognised for their crucial roles in enhancing the efficacy of pharmacological treatments, improving clinical outcomes, and alleviating the disease burden [6]. An integrative treatment strategy that combines lifestyle interventions with Guideline-Directed Medical Therapy (GDMT) presents a synergistic approach to optimising cardiovascular function, managing symptoms, and enhancing overall patient prognosis [7]. This narrative review aims to evaluate the combined impact of lifestyle modifications and pharmacological therapy in managing HF.

## Review

Literature search strategy

Relevant peer-reviewed articles and clinical guidelines were identified using the PubMed, Scopus, Cochrane Library, and Google Scholar databases. The keywords included "Lifestyle Modifications," OR "Pharmacological Treatment," AND "Heart Failure," with the search limited to publications from 2000 to 2025. Studies were selected based on methodological rigour, relevance to HF management, and their contribution to understanding the synergy between lifestyle modifications and pharmacological therapy.

Pathophysiology of HF

Mechanisms Underlying HF: Systolic vs. Diastolic Dysfunction

HF is categorised into two main pathophysiological types: systolic HF with reduced ejection fraction (HFrEF) and diastolic dysfunction with HF with preserved ejection fraction (HFpEF). HFrEF involves impaired contractility and decreased left ventricular ejection fraction (LVEF), often resulting from ischaemic heart disease, myocarditis, or dilated cardiomyopathy. In contrast, HFpEF features impaired ventricular relaxation, abnormal stiffness, and elevated filling pressures, commonly associated with hypertensive heart disease, diabetes mellitus, and obesity-related cardiometabolic dysfunction [1,8].

Role of Neurohormonal Dysregulation, Inflammation, and Oxidative Stress

Central to the pathophysiology of HF is neurohormonal dysregulation, characterised by hyperactivity of the sympathetic nervous system, activation of the renin-angiotensin-aldosterone system (RAAS), and impaired function of natriuretic peptides. These alterations result in vasoconstriction, fluid retention, myocardial hypertrophy, fibrosis, and progressive ventricular remodelling [9]. Simultaneously, chronic systemic inflammation and oxidative stress exacerbate myocardial injury and dysfunction, underscoring the necessity for interventions that modulate these pathological processes [9,10].

Impact of Comorbidities: Hypertension, Diabetes, and Obesity

Comorbid conditions significantly complicate the management of HF by exacerbating underlying pathological processes. Hypertension accelerates cardiac remodelling, diabetes contributes to metabolic disturbances in the myocardium, and obesity promotes systemic inflammation and adverse relationships between cardiac structure and function [11]. Effective management of HF requires integrated strategies that address these modifiable risk factors through targeted pharmacological therapies, along with comprehensive lifestyle change interventions.

Pharmacological treatment of HF

Overview of Pharmacological Management

Pharmacological management is essential for treating HF, aiming to alleviate symptoms, slow disease progression, and improve patient survival. Current treatment strategies incorporate various drug classes that target different pathophysiological pathways, underscoring the need for individualised, patient-centred approaches.

Renin-Angiotensin System Inhibitors

Renin-angiotensin system inhibitors (CONSENSUS trial), particularly angiotensin-converting enzyme inhibitors (ACEis), angiotensin receptor blockers (ARBs), and angiotensin receptor-neprilysin inhibitors (ARNIs), form the cornerstone of HF therapy [12]. These medications mitigate harmful neurohormonal activation by decreasing vasoconstriction, fluid retention, and myocardial fibrosis, ultimately lessening cardiac remodelling and dysfunction. The prospective comparison of ARNI with enalapril in HF (PARADIGM-HF trial) notably demonstrated the superior outcomes of enalapril compared to ACEis, indicating improved survival and reduced hospitalisation rates in HF patients [13].

Beta-Blockers

Beta-blockers significantly improve outcomes in HF patients by modulating the sympathetic nervous system. They reduce myocardial oxygen demand by lowering heart rate and contractility, which diminishes cardiac workload and shields the myocardium from excessive sympathetic stimulation. Clinical trials have consistently demonstrated their efficacy in enhancing survival rates and decreasing hospital admissions for HF patients, particularly those with HFrEF [14,15].

Aldosterone Antagonists

Aldosterone antagonists, such as spironolactone and eplerenone, effectively reduce morbidity and mortality related to HF by alleviating fluid retention and sodium overload, minimising myocardial fibrosis, and slowing the progression of ventricular remodelling. They are particularly advantageous for patients with severe symptoms or post-myocardial infarction HF, as they significantly decrease hospitalisations and mortality risks, as demonstrated in landmark trials [16].

Diuretics for Symptom Management

Diuretics, including loop and thiazide diuretics, are primarily used for the symptomatic management of HF by alleviating fluid overload. While they provide immediate relief from symptoms associated with congestion and oedema, their role in enhancing long-term survival remains limited. Due to potential adverse effects, such as electrolyte imbalances and kidney dysfunction, diuretics must be carefully managed, emphasising their adjunctive rather than foundational role in HF treatment [17].

Sodium-Glucose Cotransporter-2 (SGLT2) Inhibitors and Emerging Therapies

SGLT2 inhibitors, initially developed for managing diabetes mellitus, have recently emerged as effective therapeutic options for HF management. Clinical trials demonstrate their ability to significantly reduce HF-related hospitalisations and cardiovascular mortality, while enhancing quality of life, irrespective of diabetic status. The mechanisms underlying these benefits include diuretic effects, improved glycaemic control, favourable weight reduction, and protective cardiovascular effects, independent of glucose-lowering mechanisms [18].

Furthermore, novel therapeutic agents, such as omecamtiv mecarbil, a selective cardiac myosin activator, and vericiguat, a soluble guanylate cyclase stimulator, are currently under active investigation. Preliminary evidence from recent trials suggests that these agents offer incremental benefits by enhancing myocardial contractility and improving endothelial function, indicating their potential role in comprehensive HF management strategies [19].

Limitations of Pharmacological Treatment Alone

Despite significant advancements, pharmacological treatment alone has inherent limitations in fully addressing the complex and multifactorial nature of HF. These limitations encompass patient non-adherence due to the complexity of medications, adverse effects, limited capabilities in symptom management, and insufficient attention to lifestyle-related comorbidities, such as obesity, poor dietary choices, and a sedentary lifestyle. Consequently, there is increasing recognition of the necessity to integrate comprehensive lifestyle modifications alongside pharmacological therapies to optimise treatment outcomes and effectively meet broader patient-specific needs [19,20].

Lifestyle modifications in HF management

Dietary Interventions

Dietary interventions are crucial in managing HF by optimising cardiac function, preventing fluid overload, and enhancing overall patient outcomes. Guidelines typically recommend a low-sodium diet (below 2 gm daily) and controlled fluid intake to minimise fluid retention and cardiac strain. Despite these recommendations, there is ongoing debate regarding optimal sodium levels and their variations. Dietary patterns, such as the Dietary Approaches to Stop Hypertension (DASH), have consistently demonstrated benefits in reducing cardiovascular events and improving endothelial function, highlighting their potential to enhance HF management [21,22]. Supplementation with omega-3 fatty acids has garnered attention for its cardioprotective properties, mainly due to its anti-inflammatory effects. In contrast, evidence regarding coenzyme Q10 (CoQ10) supplementation, while promising in preliminary studies, remains inconclusive and requires further research [23].

Physical Activity and Exercise Training

Structured exercise programmes and cardiac rehabilitation significantly enhance physical capacity, cardiovascular health, and the quality of life for patients with HF [24]. Aerobic exercise, which is widely recommended, improves cardiac output, endothelial function, and exercise tolerance while reducing hospitalisation rates. In addition to aerobic activities, resistance training effectively increases muscle strength, functional status, and patient autonomy without negatively impacting ventricular performance. Recent evidence comparing high-intensity interval training (HIIT) to moderate-intensity continuous training highlights HIIT’s superior benefits in enhancing peak oxygen consumption, cardiovascular function, and cardiac remodelling [25]. However, considerations regarding HIIT, particularly training load safety, tolerability, and adherence, remain critical, especially for patients with advanced stages of the disease [21,26].

Weight Management and Obesity Control

Obesity significantly exacerbates HF by increasing cardiovascular workload, inflammation, and maladaptive cardiac remodelling [27]. Conversely, cachexia adversely impacts prognosis by leading to muscular and functional deterioration. Effective weight management strategies, including nutritional interventions and tailored exercise programmes, have proven crucial in reducing HF progression and improving patient responsiveness to pharmacological treatments. In selected cases, bariatric surgery offers significant cardiovascular benefits; however, its application is tempered by concerns regarding perioperative risks and potential nutritional deficiencies, requiring stringent patient selection criteria and a multidisciplinary care framework [11,28]

Smoking and Alcohol Cessation

Smoking significantly exacerbates HF pathophysiology due to oxidative stress, endothelial dysfunction, inflammation, and impaired myocardial oxygenation [29]. Therefore, giving up smoking is strongly advised to slow disease progression and reduce hospitalisation rates. Similarly, managing alcohol consumption is crucial, as excessive intake can lead to cardiac dysfunction, arrhythmias, and worsened HF symptoms. Adhering to the recommended limits for alcohol intake, or opting for complete abstinence, has demonstrated significant improvements in cardiac function and reduced HF-related hospitalisations [30]. Comprehensive cessation programmes that incorporate counselling and pharmacotherapy greatly enhance adherence and improve clinical outcomes [31].

Psychosocial and Behavioural Aspects

Psychosocial stressors, including depression, anxiety, stress, and social isolation, profoundly impact the management and prognosis of HF patients [32]. Psychological distress often diminishes self-care abilities, negatively affects medication adherence, and exacerbates symptom severity. Implementing cognitive-behavioural therapy (CBT) and mind-body interventions, such as yoga and meditation, has shown efficacy in alleviating depressive symptoms, enhancing self-management behaviours, and significantly improving patients' quality of life. These interventions highlight the importance of integrating psychological care into HF treatment protocols, thereby supporting overall patient resilience and promoting holistic wellness [33-35].

Synergistic effects: combining lifestyle interventions with pharmacological therapy

Rationale for a Multimodal Approach

The complexity and progressive nature of HF require an integrative, multimodal treatment approach that extends beyond pharmacological management alone. While pharmacological therapies mainly target neurohormonal mechanisms and offer symptomatic relief, they often fail to adequately address the underlying, modifiable lifestyle-related risk factors, including poor dietary habits, physical inactivity, obesity, smoking, and psychosocial stress. Therefore, it is vital to adopt an integrative approach that combines pharmacological treatment with targeted lifestyle interventions in order to achieve comprehensive management of HF, reduce disease burden, and improve patient outcomes [22,28,31].

Exercise and Beta-Blockers

Structured exercise training significantly enhances the clinical effectiveness of beta-blockers in managing HF. Regular aerobic exercise improves tolerance to beta-blockers by promoting a favourable autonomic balance, reducing the activation of the sympathetic nervous system, and enhancing cardiac efficiency. Clinical trials consistently demonstrate reductions in hospitalisations and improved quality of life among HF patients participating in exercise-based cardiac rehabilitation programmes. Notably, trials such as HF: A Controlled Trial Investigating Outcomes of Exercise TraiNing (HF-ACTION) and Optimizing Exercise Training in Prevention and Treatment of Diastolic Heart Failure (OptimEx) highlight that structured exercise significantly complements beta-blocker therapy by improving functional capacity and reducing hospitalisation rates [36,37].

Dietary Modifications and RAAS Inhibitors

Dietary sodium restriction plays a vital role in enhancing the effectiveness of RAAS inhibitors, such as ACEis, ARBs, and ARNIs. Sodium restriction amplifies the antihypertensive and cardioprotective effects of RAAS inhibitors by minimising fluid retention, reducing myocardial workload, and improving endothelial function. Furthermore, potassium-rich diets synergistically support the pharmacological action of ACEis by alleviating potential drug-induced hypokalaemia, thereby enhancing patient safety and therapeutic outcomes [38].

SGLT2 Inhibitors and Lifestyle Adjustments

SGLT2 inhibitors offer significant cardiovascular benefits, particularly when paired with lifestyle interventions focused on weight management and glycaemic control. Lifestyle modifications, such as dietary changes and increased physical activity, greatly enhance the effectiveness of SGLT2 inhibitors by improving insulin sensitivity, promoting sustainable weight loss, and reducing cardiovascular strain. Recent studies indicate improved HF outcomes and fewer hospital admissions in patients receiving combined treatment with lifestyle interventions and SGLT2 inhibitors, highlighting the importance of an integrative therapeutic approach [38-40].

Weight Loss, Metabolic Health, and Pharmacotherapy

Maintaining a healthy weight profoundly affects pharmacological efficacy in patients with HF by improving drug metabolism, enhancing therapeutic responses, and reducing adverse events. Weight loss significantly increases diuretic responsiveness, alleviating fluid retention, peripheral oedema, and hospitalisations. Case studies have demonstrated notable improvements in HF symptom management and overall patient outcomes following medically supervised weight loss interventions, underscoring the importance of integrated weight management strategies alongside conventional pharmacological treatments [41].

Mechanistic insights into synergy

Neurohormonal Modulation

Both pharmacological and non-pharmacological strategies effectively address neurohormonal dysregulation, a central pathophysiological feature of HF. Pharmacological agents, such as ACEis, beta-blockers, and aldosterone antagonists, reduce sympathetic overactivation by blocking or dampening key pathways in the RAAS and sympathetic nervous system [42]. Lifestyle interventions, such as structured exercise and stress reduction techniques (e.g., meditation and yoga), further enhance these effects by reducing chronic sympathetic activation, improving vagal tone, and promoting autonomic balance. Consequently, combined strategies synergistically normalise neurohormonal pathways, significantly enhancing cardiovascular outcomes [37,43].

Inflammation and Oxidative Stress

Inflammation and oxidative stress are pivotal in the pathogenesis and progression of HF [10]. Pharmacological therapies, such as RAAS and SGLT2 inhibitors, effectively reduce pro-inflammatory cytokines and markers of oxidative stress. Lifestyle modifications, particularly dietary interventions rich in antioxidants (e.g., the Mediterranean diet), supplementation with omega-3 fatty acids, and regular physical exercise, significantly complement these drug effects by further decreasing systemic inflammation and oxidative stress while enhancing antioxidant defence mechanisms. This integrative approach has demonstrated considerable reductions in inflammatory markers, such as C-reactive protein (CRP) and tumour necrosis factor-alpha (TNF-α), thereby alleviating myocardial damage and improving clinical outcomes [10,40].

Endothelial Function and Vascular Health

Endothelial dysfunction is a critical factor in the progression of HF and associated cardiovascular complications. Pharmacological treatments, particularly RAAS inhibitors and beta-blockers, enhance endothelial function by reducing vascular inflammation and promoting vasodilation. Concurrently, structured physical activity significantly increases endothelial nitric oxide bioavailability, improves endothelial-dependent vasodilation, and contributes to overall vascular health. Evidence from clinical studies indicates that the combined application of pharmacotherapy, alongside regular aerobic or interval training, markedly enhances endothelial function, demonstrating clear synergistic cardiovascular benefits that surpass those of monotherapy alone [44].

Myocardial Remodelling and Regeneration

**Figure 1:**
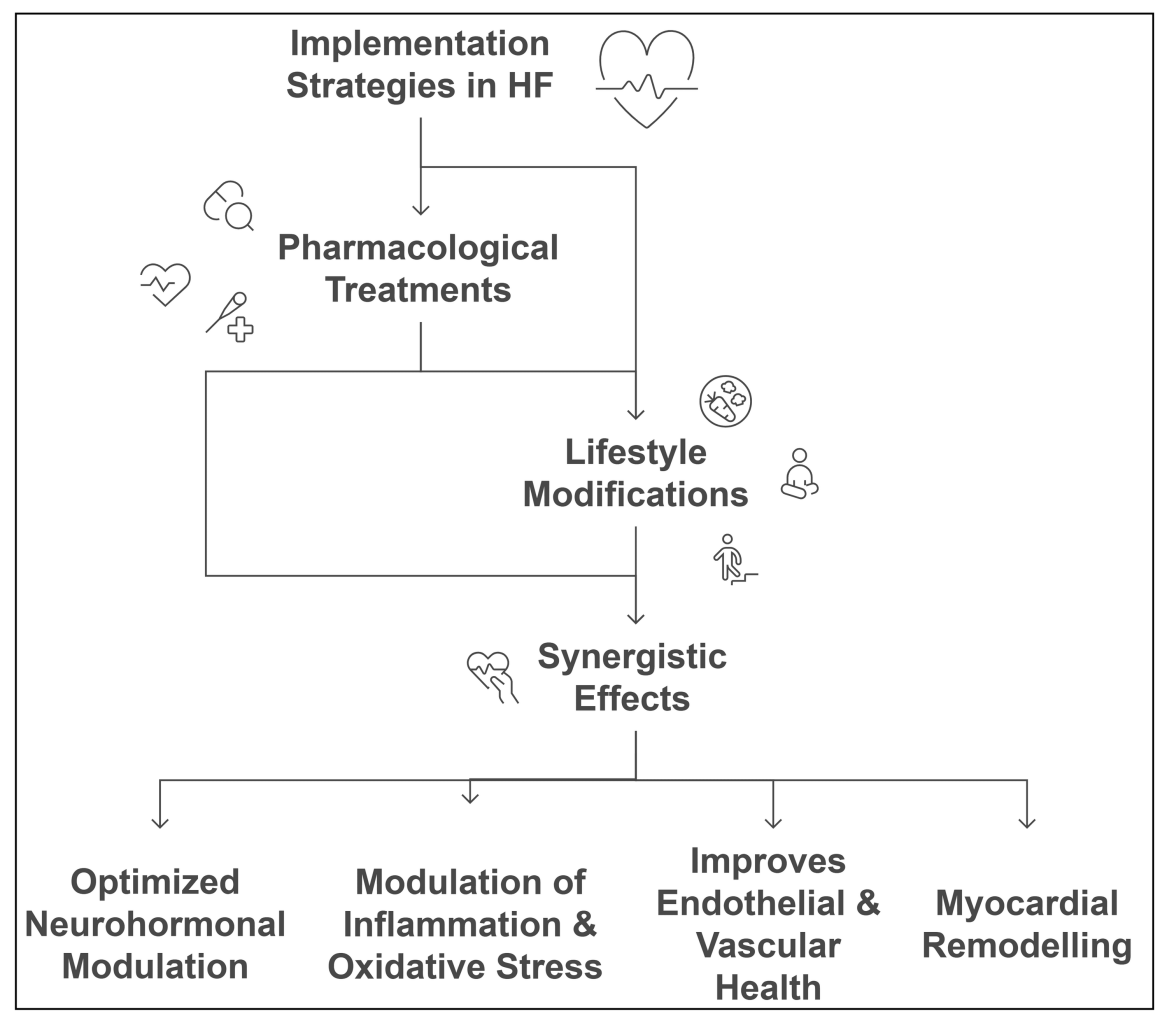
Schematic summary of synergistic impact of combination of pharmacological treatments and lifestyle modifications in HF management. Credit: This image was created by the author using PowerPoint tools (Microsoft® Corp., Redmond, WA, USA). HF, Heart failure

Myocardial remodelling, characterised by adverse structural and functional alterations in the heart, significantly impacts the prognosis of HF. Pharmacological treatments, including ARNIs, beta-blockers, and mineralocorticoid receptor antagonists, directly reduce pathological cardiac remodelling by modulating neurohormonal and inflammatory pathways. Furthermore, lifestyle interventions, primarily structured exercise and dietary modifications, work synergistically to support myocardial regeneration and delay adverse remodelling by mitigating mechanical stress, inflammation, and oxidative damage to cardiac tissues. Integrating lifestyle and pharmacological therapies provides substantial cardioprotective effects, notably enhancing cardiac function, reducing the exacerbation of HF, and prolonging patient survival [45]. An overview of the synergistic activity of pharmacological treatment along with lifestyle modifications is shown in Figure 1.

Patient adherence and real-world challenges

Importance of Patient Education

Patient education is vital to ensure the integrative approach to managing HF. Comprehensive education empowers patients with knowledge about their condition, the rationale behind therapeutic interventions, and practical strategies for self-management. Enhanced patient understanding improves medication compliance, adherence to dietary recommendations, regular physical activity, and effective management of psychosocial stressors. Educational interventions, particularly those provided through structured patient counselling and multidisciplinary teams, have significantly improved adherence, self-care behaviours, and clinical outcomes in HF populations [46].

Barriers to Implementation

Various barriers hinder the effective implementation of combined pharmacological and lifestyle interventions in HF care, including socioeconomic challenges, limited access to healthcare, and cultural factors. Socioeconomic obstacles, such as financial difficulties, inadequate insurance coverage, and restricted access to healthcare resources, significantly affect patients' adherence to recommended treatments. Additionally, cultural beliefs and practices may conflict with standard medical advice, influencing patient acceptance and long-term adherence. These barriers necessitate culturally sensitive and economically viable intervention strategies for diverse patient populations [46,47].

Strategies to Enhance Adherence

Effective strategies can improve patient adherence to integrative HF management. Behavioural interventions, including motivational interviewing and CBT, significantly improve adherence by addressing psychological barriers and bolstering patient motivation and self-efficacy. Digital health solutions, such as telemedicine platforms, mobile applications for self-monitoring, and electronic medication reminders, offer accessible, scalable, and cost-effective ways to enhance patient engagement and continuous monitoring. The CHAMPION trial demonstrated that haemodynamic-guided HF management using the CardioMEMS sensor significantly reduced hospitalisations related to HF. Additionally, it showed favourable safety outcomes, with minimal device-related complications and high system reliability over six months [48]. Furthermore, community-based programmes, which involve peer support groups, educational workshops, and local healthcare partnerships, cultivate a supportive environment that encourages sustained adherence and better health outcomes [46,49].

Clinical implications and future recommendations

Need for Multidisciplinary Teams

The complexity of managing HF underscores the necessity for a collaborative, multidisciplinary healthcare approach. Teams of cardiologists, dietitians, physiotherapists, psychologists, and specialised nursing staff provide comprehensive and coordinated care that addresses the diverse needs of HF patients. Integrating various healthcare disciplines supports holistic patient management, encourages treatment adherence, optimises therapeutic outcomes, and significantly enhances the quality of life for patients. Recent evidence highlights that multidisciplinary HF management programmes significantly reduce hospital readmissions, enhance self-care skills, and improve overall patient outcomes [49].

Personalised Treatment Plans

Personalising treatment plans is essential for maximising the efficacy of lifestyle interventions alongside pharmacological therapy. Tailoring recommendations based on individual patient characteristics, such as age, comorbid conditions, socio-cultural background, and personal preferences, greatly enhances adherence and therapeutic outcomes. Personalised interventions include customised dietary guidelines, individualised exercise prescriptions, tailored psychological support, and flexible pharmacological strategies. This patient-centred approach fosters improved engagement, adherence, and overall satisfaction, ultimately leading to better clinical outcomes in diverse HF patient populations [49].

Policy Recommendations

Integrating structured lifestyle coaching into standard HF care guidelines and policies is essential for promoting the widespread adoption of lifestyle interventions alongside pharmacological therapies. Policy-level initiatives should support the integration of lifestyle interventions into routine clinical practice, emphasising reimbursement structures for multidisciplinary care, encouraging healthcare provider training, and fostering the development of healthcare infrastructure. Furthermore, policy-driven support for community-based programmes, digital health innovations, and patient education initiatives can significantly bridge existing gaps in adherence and implementation, ultimately transforming HF management at both individual and population levels [38].

## Conclusions

An integrative approach that combines pharmacological therapies with targeted lifestyle modifications is essential for comprehensive HF management. Pharmacological treatments, including RAAS inhibitors, beta-blockers, aldosterone antagonists, diuretics, and emerging agents such as SGLT2 inhibitors, effectively address key pathophysiological mechanisms. However, incorporating lifestyle interventions, such as dietary modifications, structured physical activity, weight management, smoking cessation, and psychosocial support, significantly enhances clinical efficacy.

This approach targets modifiable risk factors and complements pharmacological treatments. This synergistic method improves patient adherence, reduces hospitalisations, enhances cardiac function, and optimises patient outcomes. This holistic, integrative treatment paradigm represents the most effective strategy to reduce the global HF burden and significantly improve patients' quality of life and survival outcomes.
